# Germinal pathogenic CHEK2, novel APC and somatic JAK2V617F variants in a young patient with colorectal cancer, atypical leukemia, cerebral tumour and aggressive course

**DOI:** 10.3332/ecancer.2025.1833

**Published:** 2025-01-23

**Authors:** Lisa Ximena Rodriguez Rojas, Jorge Andrés Olave Rodriguez, Sebastián Bonilla Navarrete, Laura Valentina Carvajal, Juan José Albán Silva, Liliana Doza Martínez, Jose Antonio Nastasi Catanese

**Affiliations:** 1 Fundación Valle del Lili, Service of Human Genetics, Cali 760031, Colombia; 2 Facultad de Ciencias de la Salud, Universidad ICESI University, Cali 760031, Colombia; 3 Fundación Valle del Lili, Center of Clinical Research, Cali 760031, Colombia

**Keywords:** hereditary cancer syndromes, CHEK2, JAK2, APC

## Abstract

Higher CHEK2 and JAK2 expression have been correlated with better survival among patients with rectal adenocarcinoma, lung squamous cell carcinoma, breast cancer, ovarian cancer and several other cancer types. It has been suggested that genome alterations due to lowered or loss of CHEK2 and JAK2 expression may exacerbate cancer progression and predict poor patient survival. In this report, we present the clinical case of a 35-year-old patient exhibiting multiple tumours, an aggressive course, whose genetic analysis revealed a germinal mutation in CHEK2 gen, somatic JAK2V617F and a germinal novel variant in Adenomatous Polyposis Coli (APC) gene of uncertain significance may account for the polyposis and medulloblastoma in the patient, given the variant's genomic location. It is also possible that two germline mutations (CHEK2 and APC) are causing two concurrent conditions in the patient with poorer clinical course.

## Introduction

CHEK2, a protein kinase that is activated in response to DNA damage, is involved in cell cycle arrest. The cancer predisposition syndrome caused by pathogenic variants in CHEK2 is characterised by an increased risk of developing colorectal, breast and prostate cancer. Although CHEK2 is considered a moderate penetrance gene, cancer risks may be considered as a continuous variable, which is influenced by family history and other modifiers [1–3]. JAK2 kinase is a member of a family of tyrosine kinases involved in cytokine receptor signaling. JAK2V617F is implicated in the genesis of some chronic myeloproliferative syndromes (MPN) such as polycythemia vera, essential thrombocythemia and idiopathic myelofibrosis [4, 5]. Although these genes are associated with distinct biological processes, there is a growing interest in exploring potential molecular and phenotypic connections between them. The potential interaction between JAK2-mediated signaling pathways and cell cycle control functions modulated by CHEK2 could shed light on shared mechanisms or synergies in the pathogenesis of certain hematologic disorders and malignant neoplasms [6]. A genome-wide association study identified germline variants in the genes CHEK2, TERT, SH2B3, TET2, ATM, PINT and GFI1B, are associated with predisposition to both age-related JAK2V617F clonal hematopoiesis in the general population and myeloproliferative neoplasms. These genes impact diverse biologic pathways such as DNA damage repair and/or tumour suppressor function (CHEK2, ATM, PINT), JAK-STAT signaling (JAK2, SH2B3), cellular aging (TERT), epigenetic regulation (TET2) and erythroid/ megakaryocyte development (GFI1B). Future research aimed at understanding the interplay between mutations in JAK2 and CHEK2 will be crucial to expand our understanding of the underlying pathophysiological processes and to develop more precise and effective therapeutic strategies [7]. JAK2V617F clonal hematopoiesis has been associated with adverse outcomes, including a higher risk of hematologic cancer and overall mortality. Furthermore, it is suggested that the identified genetic variants could be potential targets for future research and therapies in the context of clonal hematopoiesis and myeloproliferative neoplasms [6]. Turcot syndrome is an autosomal recessive condition caused by biallelic mutations in the mismatch repair (MMR) genes. It is characterised by colonic polyposis and central nervous system tumours. Conversely, monoallelic mutations in adenomatous polyposis coli (APC) cause familial adenomatous polyposis (FAP) and the brain tumour-polyposis syndrome, primarily medulloblastoma (79%), as an extracolonic manifestation of FAP [8]. In this context, we present a case of a patient exhibiting polyposis coli, colorectal cancer, atypical leukemia and cerebral tumour who carries pathogenic variants in the CHEK2 and JAK2 genes and one variant of uncertain significance (VUS) in APC.

## Case report

**Figure 1.**
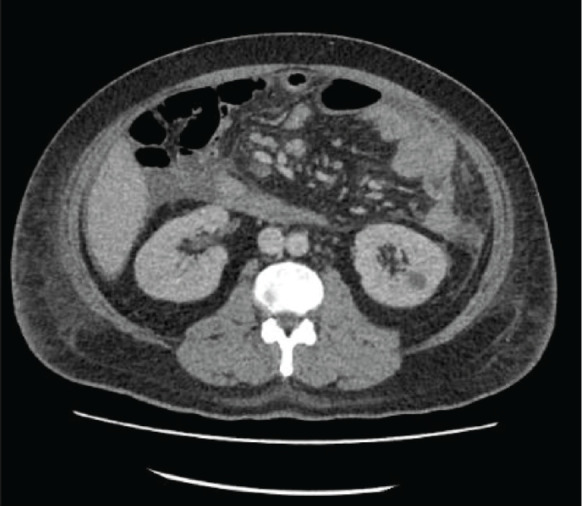
CT scan revealing a 5.6 cm neoplastic mass in the rectum and bilateral Bosniak 1 renal cysts.

**Figure 2.**
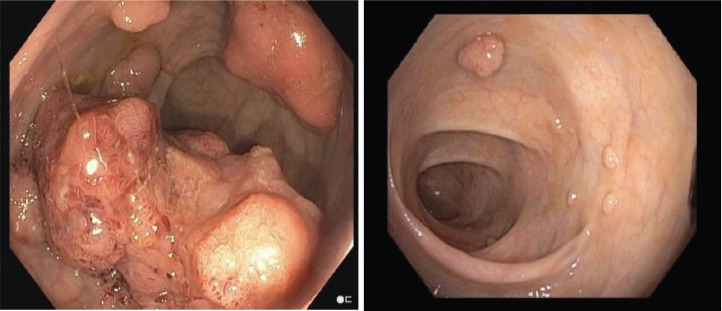
Colonoscopy, identified neoplastic mass (left) and approximately 30 sessile, adenomatous, polypoid lesions (left and right).

The patient is a 35-year-old male, born to non-consanguineous parents, with a medical history of hypertension, who presented to the emergency room with complaints of left iliac abdominal pain. A computed tomography (CT) scan was performed, revealing a 5.6 cm neoplastic mass in the rectum, located 10 mm from the anal border (Figure 1). Additionally, colonoscopy identified approximately 30 sessile, adenomatous and polypoid lesions (Figure 2). A polypectomy was performed and the histological examination confirmed the presence of tubular and tubulovillous adenomas and invasive rectal adenocarcinoma. Tumour immunohistochemistry showed intact MMR proteins and negative microsatellite instability (MSI) analysis. Polymerase chain reaction (PCR) did not detect any BRAF V600 mutation but detected a KRAS G12V mutation.

Furthermore, abdominal magnetic resonance imaging (MRI) displayed suggestive images of thrombosis in the right portal vein and left renal vein, lipomas in the pancreatic tail, prominence of the left adrenal gland, bilateral Bosniak 1 renal cysts, epidermal nodules in the anterior abdominal wall and suspicious-looking ganglia in the hepatic portal and preaortic space.

**Figure 3.**
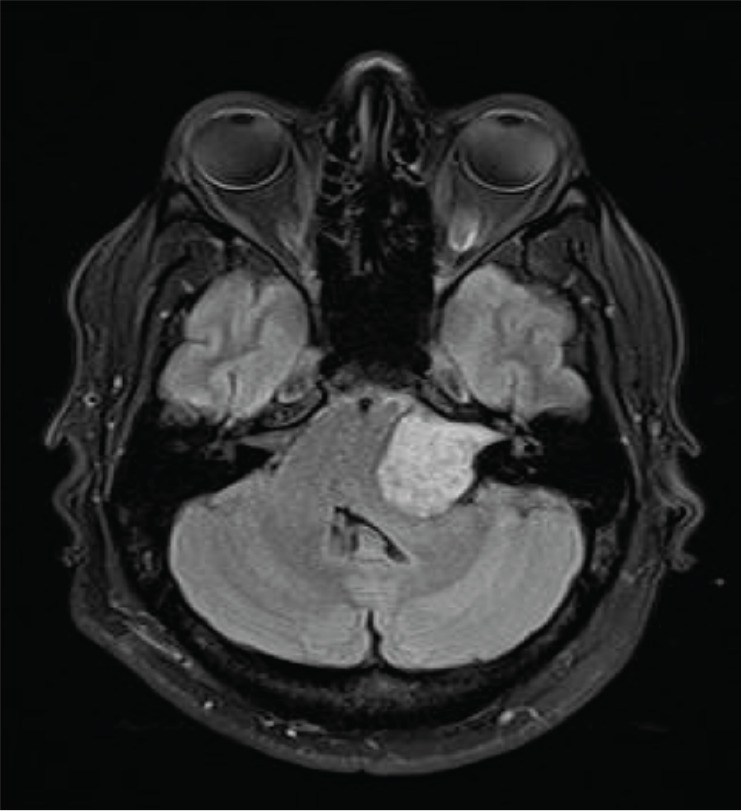
Brain MRI revealed a mass located in the posterior fossa within the cisterna of the left pontocerebellar angle.

During the hospitalisation, the patient experienced a convulsion and a brain MRI revealed a mass located in the posterior fossa within the cisterna of the left pontocerebellar angle. The mass extended to the internal auditory meatus and exerted pressure on the brainstem (Figure 3). It was not possible to obtain a biopsy of this tumour due to the patient's critical clinical condition, but its clinical appearance and imaging suggested a medulloblastoma.

A total proctocolectomy was performed, which was followed by an extended stay in the Intensive Care Unit (ICU) due to multiple infectious and hemorrhagic complications. During the ICU admission, leukocytosis and visceromegaly were observed. A diagnostic investigation was conducted, which included flow cytometry, bone marrow karyotyping, real-time PCR for bcr/abl detection and endpoint PCR for JAK2V617F. This investigation ultimately led to a diagnosis of myelodysplastic/MPN, specifically Philadelphia-negative chronic myeloid leukemia (CML).

Family history was negative, with the exception of a first-degree cousin diagnosed with colonic polyps at the age of 7, and another first-degree cousin who had uterine cancer at age 34, both without a history of consanguinity. Physical examination revealed the presence of multiple nevi on the face and upper thorax, along with a hypopigmented macule measuring 10 × 15 cm in the precordial area.

A panel for hereditary cancer of 111 genes was studied using next-generation sequencing, Genomic DNA was purified from a periferical blood sample using the Maxwell RSC Blood DNA Kit. Libraries were prepared using one enrichment-capture gene panel (OncoPlus-GeneSGKit) according to the manufacturer’s protocol. Libraries were quantified using a Qubit dsDNA HS Assay Kit and a Qubit 2.0 fluorimeter and pooled in equal volumes. Paired-end sequencing was performed using the Illumina MiSeq platform. This panel included the MMR genes (MLH1, MSH2, MSH6 and PMS2), with no MMR variants identified. The analysis of the panel included point mutations, indels, large indels, ALU insertions and copy number variations (CNVs). A heterozygous likely pathogenic (LP) variant was detected in CHEK2 c.1427C>T p.

Thr476Met, as well as a VUS in APC c.3036T>A p.Asn1012Lys. To study the Philadelphia-negative MPN, a somatic analysis of the JAK2V617F mutation was performed, which yielded a positive result. The patient experienced severe complications and ultimately passed away during hospitalisation.

## Discussion

We present a case of an adult male patient with multiple tumours, including colonic polyposis, colon adenocarcinoma, bilateral renal cysts, pancreatic lipomas, multiple abdominal nodules, a CNS mass and an atypical CML, harboring two germinal variants in CHEK2 and APC, respectively, associated with hereditary cancer predisposition. Additionally, somatic studies for his MPN showed a positive JAK2V617F.

Germinal variants in CHEK2 have been linked to tumour predisposition syndrome 4, colorectal, breast and prostate. CHEK2 is a gene that encodes for the kinase CHK2 and plays an essential role in the process of repairing double-stranded DNA breaks, as its activation in response to DNA damage prevents the cell from entering mitosis [1]. Following the recognition of DNA damage by the MRN complex and activation of ATM, CHK2 is phosphorylated by ATM, which subsequently phosphorylates p53, contributing to p53-dependent cell cycle arrest in G1 [1, 3].

The CHEK2 variant identified in exon 13 in our patient, and classified as LP, results in the substitution of the amino acid Threonine with Methionine at codon 476 of the CHEK2 protein, which is located in a moderately conserved residue within the kinase domain of the protein. *In-vivo* yeast-based functional analyses have been conducted to assess the effect of this variant on DNA damage repair [2, 9]. Roeb *et al* [10] classified the variant as damaging, while Delimitsou *et al* [9] observed an intermediate effect. This particular variant has also been identified in multiple patients with colorectal cancer [11–14], as well as breast, prostate, pancreatic and endometrial cancers [15–18]; then, functional assays on this variant have shown discordant results [19]. This is in line with the difficulties classifying CHEK2 variants, having been recognised as one of the genes with more conflicting interpretations in hereditary cancer [20]. Specifically, in colorectal cancer, there is not yet consistent evidence that CHEK2 variants contribute greatly to cancer predisposition, although a low-to-moderate risk has been found for c.1100delC and a low risk for p.I157T carriers [2].

Another germline variant found in our patient classified as a VUS was in the APC gene. APC is a tumour suppressor gene located in the long arm of chromosome 5 that is highly implicated in the pathogenesis of colorectal cancers. Genetic alterations in APC lead to activation of WNT signaling and deregulation of cell proliferation and growth, among other processes [21, 22]. Germinal variants in APC lead to multiple phenotypes, all now recognised to be a part of the FAP spectrum. These include FAP, attenuated FAP, gastric adenocarcinoma and proximal polyposis of the stomach, Gardner syndrome, lipomas and fibromas. Approximately half of these patients will have developed colorectal adenomas at age 15, and almost all by age 35. Malignant transformation risk is very high if left untreated. Extracolonic manifestations include CNS tumours and hepatobiliary, pancreatic and adrenal malignancies, lipomas and increased skin pigmentation [22]. The APC variant in our patient is a novel variant that causes a missense change in exon 15 of the gene, resulting in the substitution of the amino acid Asparagine with Lysine at codon 1012 of the APC protein. This variant has not been previously described, is not present in gnomAD samples and is predicted to be pathogenic by 9 *in-silico* predictors (Primate AI, LIST-S2, LRT, EVE, MetaLR, DANN, FATHMM-MKL, MutationTaster, CADD). Our patient presented with a diverse array of tumoural manifestations, (Table 1) including colonic polyposis, colon adenocarcinoma, atypical CML (ph-), abdominal masses soft-tissue, CNS tumour, lipomas and increased skin pigmentation. All these clinical manifestations can be caused by pathogenic variants in the APC gene. We propose that this case helps to clinically reclassify this variant as LP. It was not possible to study the variant in the APC gene in the unaffected siblings/relatives or in the affected cousin with polyps, which would have supported the reclassification of the variant as potentially pathogenic if present in those affected with polyps. It is worth noting that the patient had a medulloblastoma, and the APC VUS is located in the gene region linked to a higher risk of brain cancer, specifically medulloblastoma [8]. Our group has reported this novel variant in ClinVar as probably pathogenic in connection with this case. On the other hand, the variant in the CHEK2 gene would be related to the patient's colorectal cancer. We hypothesise that the variants found in CHEK2 and APC may explain some of the patient's phenotype, but the exact contribution of each variant to tumourigenesis remains uncertain. Additionally, the presence of the JAK2 mutation contributes to the patient's phenotype with venous thrombosis and MPN. According to recent reports, the presence of this somatic mutation along with germline variants is associated with an aggressive course of the disease [6], as occurred in the present case. The role of CHEK2 in germline predisposition to cancer is still a topic of debate, and further investigations are needed to determine its precise involvement in *in-vivo* carcinogenesis. We suggest that we are dealing with a case involving two germline mutations in the CHEK2 and APC genes, with neoplastic manifestations associated with both genes, especially with APC, and aggravated by the presence of JAK2V617F. To advance our comprehension of the underlying pathophysiological mechanisms and to develop more accurate and effective therapeutic approaches, it is essential for future research to investigate the interaction between mutations in JAK2 and CHEK2. The presence of JAK2V617F clonal hematopoiesis has been linked to negative outcomes, such as an increased risk of hematologic cancer and higher overall mortality. Additionally, it is proposed that the identified genetic variations may serve as potential targets for future investigations and treatments in the realm of clonal hematopoiesis and myeloproliferative neoplasms.

## Conflicts of interest

The authors have no interests to disclose.

## Informed consent

Informed consent was signed by the patient, and the study was approved by the ethical committee of Fundación Valle del Lili.

## Author contributions

All authors contributed to all aspects of the manuscript. Dr. Rodriguez is the corresponding author for this case report.

## Availability of data and materials

The information was extracted from the patient's medical records located in the software of Fundación Valle del Lili (SAP). The photos were taken of the patient with his prior consent for use in this specific article.

## Figures and Tables

**Table 1.** Detected mutations and their contribution to the patient's phenotype in the case.

	Mutation	ACMG classification	Origin	OMIM phenotype	Phenotype: clinical presentation
CHEK2	c.1427C>T p.Thr476Met T476M	LP ACMG CRITERIA: PP5, PS3, PM5, PM1, PS4, PM2	Germinal	Tumor predisposition syndrome	Colorectal cancer
				Colorectal cancer	
				Breast cancer	
				Prostate cancer	
Novel APC	c.3036T>A p.Asn1012Lys N1012K	VUS ACMG CRITERIA: PP5, PM2, BP1	Germinal	Brain-tumor polyposis syndrome	Poliposis coli, colorectal cancer, cerebral tumor, lipomas, skin pigmentation, abdominal soft-tissue masses
				APC	
				Gardner syndrome	
				Colorectal cancer	
				Gastric adenocarcinoma	
JAK2	c.1849G>T p.Val617Phe V617F	Pathogenic ACMG CRITERIA: PS3, PP5, PM5, PM1, PP3	Somatic	Budd-Chiari syndrome	Venous thrombosis, MPN, MCL ph(-)
				Thrombocythemia	
				Myelofibrosis	
				Acute myeloid leukemia	
				MPN	
